# Association between smoking and risk of death in patients with sepsis: A systematic review and meta-analysis

**DOI:** 10.18332/tid/150340

**Published:** 2022-07-15

**Authors:** Nai Zhang, Yujuan Liu, Chuang Yang, Peng Zeng, Tao Gong, Lu Tao, Xinai Li

**Affiliations:** 1 Department of Emergency, Jiangxi Province Hospital of Integrated Chinese and Western Medicine, Nanchang, China; 2 Department of Respiratory Medicine, Jiangxi Province Hospital of Integrated Chinese and Western Medicine, Nanchang, China

**Keywords:** meta-analysis, smoking, mortality, sepsis, follow-up period

## Abstract

**INTRODUCTION:**

Although some research papers have suggested that smoking may increase mortality in patients with sepsis, no evidence has been produced in this regard. This systematic research evaluated the risk of death in patients with sepsis who were smokers to facilitate better clinical decision making.

**METHODS:**

This is a systematic review registered in PROPERO (CRD42022296654). Searches were conducted to identify suitable studies from the databases of PubMed, Embase, Web of Science and the Cochrane Controlled Register of Trials from January 1980 to June 2021. Two independent reviewers screened the articles using keywords and extracted the data. The Newcastle–Ottawa Scale (NOS) was used to evaluate the quality of evidence. The primary endpoints included the mortality of patients with sepsis.

**RESULTS:**

Five studies involving 2694 participants were included in our study. Among the five included articles, three studies had an NOS score of 6, while the other two had an NOS score of 7. The results showed that a significantly higher risk of death was observed in smokers with sepsis compared with non-smokers with sepsis (hazard ratio, HR=1.62; 95% CI: 1.11–2.37, p=0.01). Among the patients followed for more than 2 months, the mortality rate of smokers was significantly higher (2.33 times) than that of non-smokers (HR=2.33; 95% CI: 1.83–2.96, p<0.01). The difference in mortality did not reach statistical significance when the follow-up period was shorter than 2 months (HR=1.22; 95% CI: 0.96–1.56, p=0.10).

**CONCLUSIONS:**

Smoking increased mortality in patients with sepsis when the follow-up period was longer than 2 months.

## INTRODUCTION

The tobacco-smoking epidemic is a major public health problem impacting global health^1^. Tobacco smoking is thought to contribute to more than 7 million deaths annually worldwide^2,3^. Furthermore, smoking increases the incidence and mortality of various diseases, including cardiovascular and lung disorders^4-6^. Smoking-related diseases are estimated to account for approximately 5.7% of global health expenditure, placing a huge economic burden on individuals and healthcare systems worldwide^7,8^.

Sepsis is a life-threatening organ injury that is induced by an abnormal immune response to microbial infection^9^. About 48.9 million sepsis cases were reported globally in 2017, contributing to 19.7% of all deaths worldwide^10^. The COVID-19 pandemic has increased the cases of sepsis further^11^. The Health Outcome Predictive Evaluation for COVID-19 Registry reported that smokers were at high risk of developing sepsis in COVID-19. In addition, sepsis was associated with a high mortality rate in COVID-19^12^. Furthermore, sepsis was observed more frequently in overweight and obese patients than in those without excess weight or obesity in COVID-19^13^.

Severe acute respiratory syndrome coronavirus 2 (SARS-CoV-2) causes an uncontrolled inflammatory response in patients, with severe new coronary pneumonia characterized by a significant release of pro-inflammatory cytokines, leading to lymphocyte dysfunction and abnormalities of granulocytes and monocytes^14^. Although the treatment of sepsis has developed rapidly in the past few years, the mortality rate from sepsis is still increasing, resulting in a significant burden on all healthcare systems^15^.

Studies have shown that the risk of death is increased in patients with sepsis who are elderly^16^, have a history of cancer^17^, chronic liver disease^18^, acute kidney injury^19^, multiple organ failure^20^ and human immunodeficiency virus infection^21^. However, given the complexity of the effects of that, it is difficult to elucidate its true footprint. Some research articles have suggested that smoking may up-regulate inflammatory factors^22-24^ and increase mortality in patients with sepsis^25-27^. However, no evidence has been produced on the association between smoking and the risk of death in patients with sepsis.

To clarify this argument, we conducted a systematic review using a meta-analysis to compare the mortality rates between smokers and non-smokers in patients with sepsis and assess the mortality-related risk from sepsis among current smokers.

## METHODS

### Search strategy

This study was conducted following the Preferred Reporting Items for Systematic Reviews and Meta-Analysis guidelines^28^ (registration information: PROSPERO CRD42022296654). The databases of PubMed, Embase, the Web of Science and the Cochrane Controlled Register of Trials from January 1980 to June 2021 were reviewed. This search was supplemented with a manual screening of retrospective studies. The references were searched manually to identify additional relevant manuscripts using the following keywords: ‘Sepsis’, ‘Bloodstream Infection’, ‘Pyemia’, ‘Septicemia’, ‘Poisoning’, ‘Blood Poisonings’, ‘Severe Sepsis’, ‘Smoking’, ‘Behavior’, ‘Smoking’, ‘death’, ‘mortality rate’ and ‘models, statistical’. Before the final analyses, the search was repeated to identify additional studies.

### Selection criteria

The inclusion criteria were as follows: 1) Studies evaluating the mortality in patients with sepsis who smoked, using hazard ratios (HRs), odds ratios (ORs) or relative risk ratios (RRs) with a corresponding 95% confidence interval (CI); and 2) A current smoker was characterized by having smoked at least 100 cigarettes in their lifetime, while non-smokers had smoked less than 100 cigarettes in their lifetime^5^. Sepsis was graded using different versions of the sepsis guidelines, i.e. sepsis version 1^29^, sepsis version 2^30^, and sepsis version 3^31^.

The following were excluded from this systematic review: reviews, letters, comments, studies of pediatric patients, studies of animals, and studies not published in English.

### Data extraction

Two investigators independently collected the first author’s name, the study design, the publication year, the study location, the sample size, the smoking habits, the duration of the follow-up and the sepsis events as well as the HRs, ORs and RRs and their associated 95% CIs or standard errors. Any disagreements were resolved through discussion with a third investigator.

### Quality assessment

The quality of the included studies was assessed using the Newcastle–Ottawa Scale (NOS)^32^. The highest score was 9 points, which was based on three items: comparability (2 points), selection (4 points) and outcome (3 points). Low-quality studies scored <6 points, medium-quality studies scored 6–7 points, and high-quality studies scored 8–9 points.

### Statistical analysis

The data on mortality were analyzed using HRs. The inverse-variance weighted random-effects method was used to aggregate the HRs and 95% CIs of all the included studies. The I^2^ test^33^ and the chi-squared test were used to assess the heterogeneity across the studies. A fixed-effects model was used in cases of low heterogeneity (I^2^<50%, or p>0.1). Otherwise, a random-effects model was used. A Galbraith radial plot was produced to identify the potential causes of heterogeneity, while a Forest plot calculated the aggregated relative risk of death from all studies. A sensitivity analysis was performed to determine whether an individual study had any impact on the aggregated results of the meta-analysis. A funnel plot analysis together with Egger’s test determined publication bias. A meta-regression evaluated potential covariates linked with sepsis. Potential variables between studies that may have had an impact on the results of this research were also evaluated.

Based on these findings, an additional subgroup analysis was conducted to evaluate how these variables impacted the HRs. Statistical analyses were conducted using Stata (version 14.0) software. A p<0.05 was considered statistically significant.

## RESULTS

### Search results

**Figure 1:**
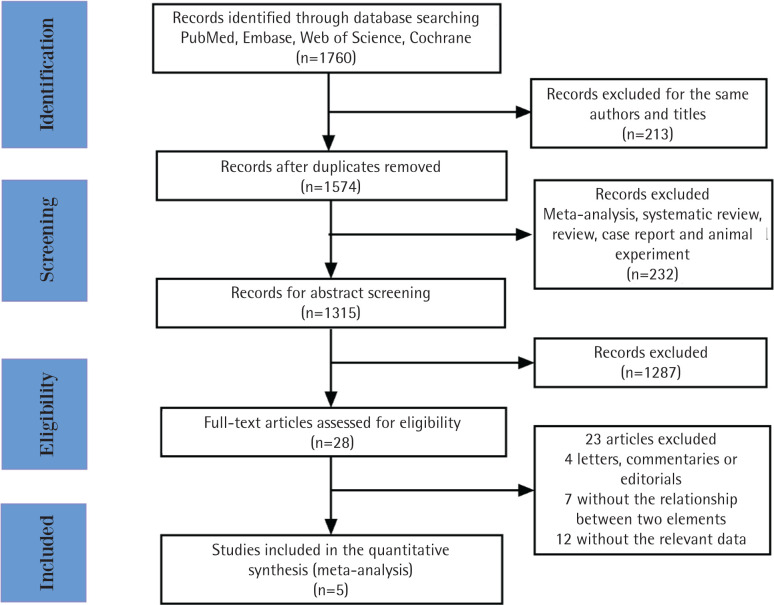
Flowchart of the study

A total of 1760 relevant studies were initially retrieved. After removing duplicates, 1547 studies were additionally screened. A total of 232 studies were excluded that consisted of meta-analyses, systematic reviews, reviews, case reports and animal experiments. Among the remaining 1315 articles, 1287 studies were excluded after the abstracts were screened. The remaining 28 studies were evaluated, and 23 studies were eventually excluded, 4 of which were letters, commentaries, or editorials; 7 did not report any investigation between smoking and the mortality in sepsis, and the remaining 12 studies did not report risks associated with current smoking and mortality in sepsis (Figure 1). Finally, 5 studies were included in our research. Among the 5 included articles, 3 studies had an NOS score of 6, while the other 2 studies had an NOS score of 7. All studies had an NOS score of 6 or higher, indicating good quality (Table 1).

**Table 1:** NOS scores

*Study*	*Selection*	*Comparability*	*Outcome*	*NOS score*	*Definition of sepsis used*
Pittet^34^ 1993	★★	★★	★★	6	Septicemia was defined as a clinical condition associated with one or more positive blood cultures for a commonly accepted pathogen or two or more positive blood cultures for less usual pathogens (coagulase negative staphylococci and candida spp) and either signs of severe infection or evidence of systemic response to severe infection
Salive^35^ 1993	★★	★★	★★★	7	Septicemia was defined as a clinical condition associated with one or more positive blood cultures for a commonly accepted pathogen or two or more positive blood cultures for less usual pathogens (coagulase negative staphylococci and candida spp) and either signs of severe infection or evidence of systemic response to severe infection
Alroumi^25^ 2018	★★★	★★	★	6	The sepsis-2 consensus definitions were utilized
Kempker^26^ 2018	★★	★★	★★	6	Sepsis is defined as a dysregulated immune response to infection that results in acute organ dysfunction
Pehlivanlar^27^ 2021	★★★	★★	★★	7	Sepsis is defined as life-threatening organ dysfunction caused by a dysregulated host response to infection

### Characteristics of the selected studies

Five studies involving 2694 participants were included in this research. Among the included studies, four^25,26,34,35^ were retrospective, and one^27^ was a prospective observational study. The publication dates ranged between 1993 and 2021, and the sample sizes varied from 173 to 1523 participants. The follow-up period varied from 20 days to 6 years. All studies included both genders and clearly defined the current smoking and non-smoking populations.

### The meta-analysis of mortality

**Figure 2:**
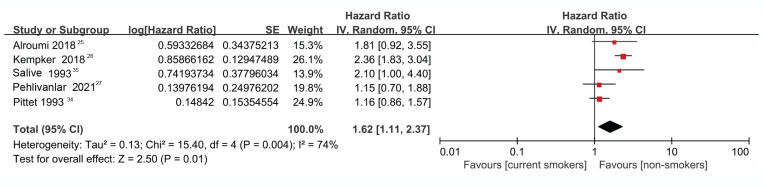
Forest plot regarding the risk of death between smokers vs non-smokers with sepsis

**Figure 3:**
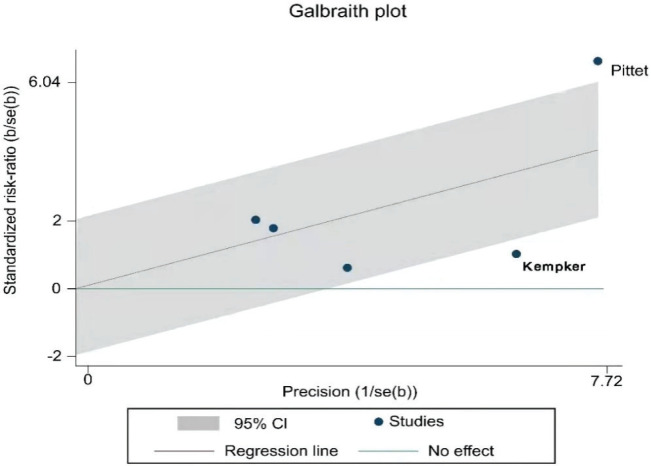
Galbraith radial plot of the included studies

The result of the heterogeneity test showed moderate to high heterogeneity (I^2^=74.0%, p=0.004) among the studies (Figure 2). Further analysis using a Galbraith radial plot (Figure 3) revealed a higher possibility of heterogeneity between Pittet et al.^34^ and Kempker et al.^26^.

Based on the findings of this analysis, we concluded that the evaluated studies had a moderate level of heterogeneity and thus, the random-effects model was used.

As shown in Figure 2, compared with non-smokers, higher mortality was found among the current smokers (HR=1.62; 95% CI: 1.11–2.37). The overall effect (Z) was 2.50 (p=0.01), indicating a significant difference between groups (Figure 2).

### Sensitivity analysis and funnel plot analysis

The analysis of mortality revealed significant heterogeneity. The sensitivity analysis showed that removing each study did not affect the stability or reliability of the results.

**Figure 4:**
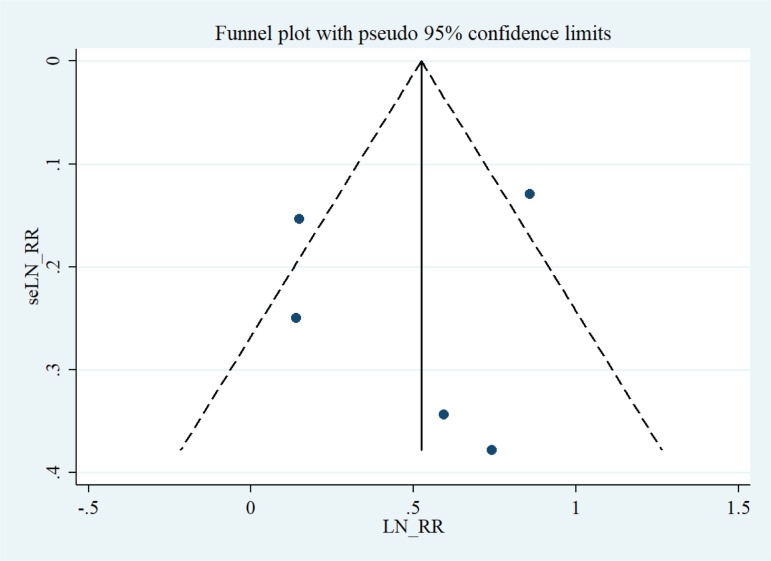
Funnel plot analysis to detect publication bias

A visual evaluation of the funnel plots revealed a symmetrical distribution (Figure 4). Accordingly, Egger’s regression asymmetry tests showed no significant bias among the studies (p=0.771).

### Meta-regression analysis to evaluate the cause of the heterogeneity

After evaluating all potential covariables, inconsistencies in the follow-up period between studies were identified as a potential key factor leading to heterogeneity. A meta-regression analysis was performed to confirm this hypothesis. The findings of the meta-regression confirmed that the follow-up period had a significant impact on effect size (p=0.034). Therefore, a further subgroup analysis was performed to investigate how the follow-up period affected mortality.

### Subgroup meta-analysis

The five studies were divided into two subgroups. Group 1 included studies with a follow-up longer than 2 months, and Group 2 included studies with a follow-up shorter than 2 months.

**Figure 5:**
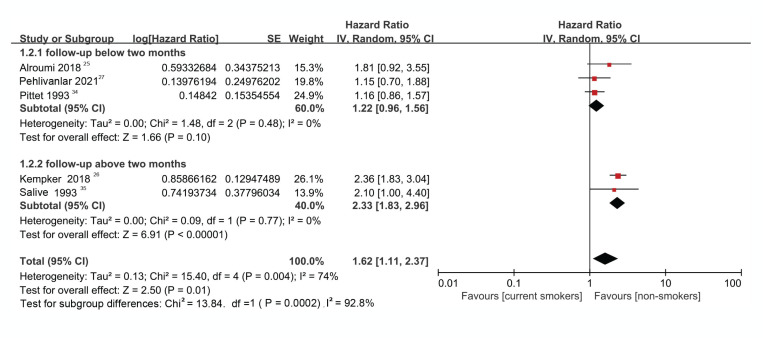
Forest plot evaluating mortality from sepsis between current smokers and non-smokers in studies with a follow-up above and below two months

There was no heterogeneity in the subgroup analysis irrespective of whether the study had a follow-up shorter than two months (I^2^=0%, p=0.48) or more than two months (I^2^=0%, p=0.77). However, there was moderate heterogeneity (I^2^=74.0%, p=0.004) when all studies were analyzed. The above analysis indicated that the follow-up period was the underlying reason for the heterogeneity between studies and thus confirmed the hypothesis that a subgroup analysis based on the follow-up period was appropriate (Figure 5).

### Forest plot analysis

For the study group with a follow-up period shorter than 2 months, the HR was 1.22 (95% CI: 0.96–1.56), indicating that the risk of death in current smokers with sepsis was 1.22 times higher than in non-smokers. However, this risk estimate was not significant (Z=1.66, p=0.10) (Figure 5). Conversely, for the study group with a follow-up period longer than 2 months, the HR was 2.33 (95% CI: 1.83–2.96). The effect size was statistically significant (Z=6.91, p<0.00001), meaning that the mortality rate of current smokers with sepsis was 2.33 times (significantly) higher than that of non-smokers (Figure 5).

### Funnel plot analysis

A visual evaluation of the funnel plots revealed a symmetrical distribution. Egger’s test showed no significant bias among the studies with a follow-up period shorter than 2 months (p=0.42) and studies with a follow-up period longer than 2 months (p=0.197).

## DISCUSSION

Sepsis affects more than 1 million people in the United States each year and has a high mortality rate. The effects of smoking on immune function are well documented, but the overall impact of smoking on the clinical manifestations of sepsis is unclear. In this study, we demonstrated the findings of our first meta-analysis, which was aimed at evaluating the association between smoking and mortality in patients with sepsis. Our findings indicate that smoking increases the mortality risk from sepsis.

The mortality rate in patients with sepsis ranges from 25% to 30%, eventually resulting in considerable healthcare costs^36,37^. Previous studies have shown that smokers have an increased risk of developing sepsis-related organ dysfunction^38^ and septic shock^39^. However, there are few relevant clinical studies, and the data are not comprehensive. This results in insufficient evidence that smoking increases mortality in patients with sepsis. Therefore, it is necessary to understand and explore whether smoking increases the risk of death in patients with sepsis.

Many previous studies have researched the effect of smoking on patients with respiratory infections. Evidence shows that smoking is related to increased mortality in patients with respiratory infections, particularly pneumonia and influenza^40,41^. Furthermore, cigarette smoking markedly induces inflammation and alters immune signaling pathways. Studies have shown that smoking can promote the body’s inflammatory response by increasing the release of pro-inflammatory factors^42-44^. In addition, smoking leads to oxidative stress, which induces systemic inflammation. Smoking is also associated with immunosuppressive effects that reduce a patient’s resistance to infection^45^. Compared with non-smokers, current smokers with sepsis are more likely to require intensive care treatment since their sequential organ failure assessment score, which is used to evaluate the severity of sepsis and predict the outcomes of patients with sepsis, tends to be higher^40,46^. However, the direct relationship between smoking and mortality in patients with sepsis remains unclear.

We also performed a subgroup analysis that considered the duration of the follow-up period. The mortality rate among smokers was 2.33 times (significantly) higher when compared with non-smokers for patients with a follow-up period longer than 2 months. However, the difference was not significant for patients with a follow-up shorter than 2 months.

Although both early and late stages of sepsis are affected by other health conditions, including chronic diseases, low socioeconomic status and multiple comorbidities^26^, the initial condition of patients with sepsis is unstable and easily deteriorated in the early stage. In addition, current smokers tend to have worse health habits (such as drinking alcohol) and are less likely to participate in influenza and pneumococcal vaccination programs^47-49^. A few studies have found alcohol abuse to be an independent predictor of mortality in multivariable analyses^47,48^, although when combined with smoking, it may increase the mortality of sepsis. Our research provides good inspiration for the clinical treatment of patients with sepsis. For patients with sepsis who like to smoke, it is important to prohibit the patient from continuing to smoke during and after treatment.

### Strengths and limitations

Our meta-analysis has some limitations that must be acknowledged. The majority of the studies used earlier versions of the sepsis guidelines for grading, and only one of the included studies used the latest version. Our results were based on unadjusted estimates; therefore, future studies should evaluate the impact of other known confounding variables, such as gender, age, body mass index and lifestyle, on the risk of mortality from sepsis among smokers. Furthermore, publication bias could have been introduced by the limited number of studies included, even though our statistical tests showed a low risk of bias. The publication bias could have been increased further by the fact that we included only studies published in English. The number of cigarettes smoked daily may also have an impact on the risk of mortality from smoking. In addition, we did not evaluate former smokers and their risk of mortality from sepsis. As a result, further studies are required. This strategy of grouping the participants into two categories is acceptable, but if options are available to include only non-smoking individuals as non-smokers, that would be more suitable.

However, there are many advantages to this study. Two researchers independently assessed the quality of the included studies using the NOS, and publication bias was evaluated by a funnel plot analysis together with Egger’s test, which provided criteria to evaluate the methodological quality of the studies. To accurately identify the relationship between sepsis and smoking, heterogeneity and sensitivity of the results were analyzed in this study.

## CONCLUSIONS

Current smokers with sepsis have a higher risk of death when the follow-up period is longer than 2 months. Although patients with a shorter follow-up period also showed an increased risk of death, the finding was not statistically significant. Thus, current smoking might be a modifiable risk in patients with sepsis. Due to the limitations of this study, further research is required.

## Data Availability

The data supporting this research are available from the authors on reasonable request.
